# MicroRNAs and cell proliferation in head and neck squamous cell carcinoma

**DOI:** 10.1186/1753-6561-7-S2-P25

**Published:** 2013-04-04

**Authors:** Flavia M Andreghetto, Maria FG Klingbeil, Renata M Soares, Décio S Pinto-Junior, Mônica B Mathor, Fábio D Nunes, Patricia Severino

**Affiliations:** 1 Albert Einstein Research and Education Institute, Albert Einstein Hospital, São Paulo, SP, Brazil; 2 School of Dentistry, Department of Oral Pathology, University of São Paulo, São Paulo, SP, Brazil; 3 Radiation Technology Center – Institute for Energy and Nuclear Research São Paulo, SP, Brazil

## Background

Functional *in vitro* studies are essential for the understanding of the role of microRNAs, small noncoding RNA molecules that function as posttranscriptional regulators, in cancer. In this study we investigated the effect of the over-expression of microRNAs previously identified as deregulated in squamous cell carcinomas of the head and neck (miRNA-1, miRNA-7, miRNA-10b e miRNA-196a) in cell proliferation.

## Materials and methods

For this purpose we performed gain-of-function assays for microRNAs in an oral squamous cell cancer cell line as well as in normal keratinocytes derived from primary cultures and evaluated the expression of validated and predicted gene targets for these microRNAs. We then evaluated the effect of the over-expression on cell proliferation by means of Ki67 staining.

## Results

Significant differences in results were seen between the cell lines and normal keratinocytes. Down-regulation of gene targets was not observed at all instances, an expected result since genes might be regulated by more than one microRNA, and by genetic and epigenetic mechanisms which are not common among all cell types. We were able to detect down-regulation of *KLF4*, a miR-10b target; *ANXA1*, a validated target,and *PTK9*, a predicted target of miR-196a were also down-regulated upon over-expression of the respective microRNA regulators in the oral cancer cell line. Different results were observed for keratinocytes, in which we confirmed down-regulation of miR-7 targets *mTOR* and *PI3KCD*, of *p70S6K1*, a miR-196a predicted target, and of the miR-10b target *HOXD10*. Over-expression of miR-7, miR-10b and miR-196a clearly interfered with cell cycle progression, but this effect was significantly stronger in keratinocytes.

## Conclusions

The expression levels of the target genes studied here could collaborate to the effect on cell proliferation, since they have all been previously related to this process. The role of microRNAs on gene regulation clearly depends on the cell type.

## Financial support

FAPESP, HIAE.

